# TGF-β1 and serum both stimulate contraction but differentially affect apoptosis in 3D collagen gels

**DOI:** 10.1186/1465-9921-6-141

**Published:** 2005-12-02

**Authors:** Tetsu Kobayashi, Xiangde Liu, Hui Jung Kim, Tadashi Kohyama, Fu-Qiang Wen, Shinji Abe, Qiuhong Fang, Yun Kui Zhu, John R Spurzem, Peter Bitterman, Stephen I Rennard

**Affiliations:** 1 Department of Internal Medicine, University of Nebraska Medical Center, Omaha, Nebraska, USA; 2 Seoul Adventist Hospital and WonKwang University Sanbon Medical Center, Seoul, Korea; 3 Department of Respiratory Medicine, Graduate School of Medicine, University of Tokyo, Tokyo, Japan; 4 Department of Respiratory Medicine, West China Hospital, West China Medical School Sichuan University, Chengdu, Sichuan P.R. China; 5 The 4^th ^Department of Internal Medicine, Nippon Medical School, Tokyo, Japan; 6 Department of Pulmonary and Critical Care Medicine, The First Hospital of Tsinghua University, Beijing, P.R. China; 7 Department of Respiratory Diseases, Jincheng Hospital, Lanzhou, P.R. China; 8 University of Minnesota, Minneapolis, Minnesota, USA

**Keywords:** transforming growth factor-beta, apoptosis, gel contraction, fibrosis, wound repair

## Abstract

Apoptosis of fibroblasts may be key for the removal of cells following repair processes. Contraction of three-dimensional collagen gels is a model of wound healing and remodeling. Here two potent inducers of contraction, TGF-β1 and fetal calf serum (FCS) were evaluated for their effect on fibroblast apoptosis in contracting collagen gels. Human fetal lung fibroblasts were cultured in floating type I collagen gels, exposed to TGF-β1 or FCS, and allowed to contract for 5 days. Apoptosis was evaluated using TUNEL and confirmed by DNA content profiling. Both TGF-β1 and serum significantly augmented collagen gel contraction. TGF-β1 also increased apoptosis assessed by TUNEL positivity and DNA content analysis. In contrast, serum did not affect apoptosis. TGF-β1 induction of apoptosis was associated with augmented expression of Bax, a pro-apoptotic member of the Bax/Bcl-2 family, inhibition of Bcl-2, an anti-apoptotic member of the same family, and inhibition of both cIAP-1 and XIAP, two inhibitors of the caspase cascade. Serum was associated with an increase in cIAP-1 and Bcl-2, anti-apoptotic proteins. Interestingly, serum was also associated with an apparent increase in Bax, a pro-apoptotic protein. Blockade of Smad3 with either siRNA or by using murine fibroblasts deficient in Smad3 resulted in a lack of TGF-β induction of augmented contraction and apoptosis. Contraction induced by different factors, therefore, may be differentially associated with apoptosis, which may be related to the persistence or resolution of the fibroblasts that accumulate following injury.

## Background

The development of fibrosis is thought to share a number of important features with normal wound repair. Both fibrosis and wound repair are characterized by the recruitment and activation of fibroblasts that differentiate to myofibroblasts [1-3]. These cells accumulate within tissue, produce extracellular matrix and remodel the local environment. Both fibrotic tissues and normal healing wounds are also characterized by myofibroblast contraction of extracellular matrix. Fibrosis, however, differs from normal wound healing in a number of important respects. Prominent among these, normal wound healing is characterized by the eventual resorption of much, if not all, of the excess connective tissue matrix and mesenchymal cells that characterize the healing phase [4]. In fibrosis, in contrast, normal tissue structures are permanently disrupted by excessive fibrotic material.

The three transforming growth factor-beta (TGF-β) isoforms are members of a family of signaling molecules [5]. TGF-β1 is believed to be a key factor in mediating both mesenchymal cell participation in wound repair and in a number of pathologic settings in fibrosis [6]. TGF-β is a potent activator of fibroblasts, inducing their differentiation into myofibroblasts and stimulating their production of extracellular matrix [7,8]. In *in vitro *experiments, TGF-β has been reported to inhibit fibroblast/myofibroblast apoptosis [9,10]. These *in vitro *experiments, however, have evaluated fibroblasts in monolayer culture. Culture of fibroblasts in three-dimensional collagen gels has been used as a system that more closely resembles tissues undergoing repair. These observations, therefore, raise an interesting and potentially important question: What would be the effect of TGF-β on the apoptosis of fibroblasts in three-dimensional collagen gel culture? Augmentation of contraction and in addition to apoptosis might lead to the net accumulation of contracted connective tissue and hence be a mechanism for the development of fibrosis.

TGF-β1 stimulates fibroblast contraction of extracellular collagenous matrices [11,12]. Interestingly, fibroblasts in a contracting matrix have been reported to undergo apoptosis [13,14]. The degree of apoptosis, moreover, has been associated with the degree of contraction in several studies [13-15]. The current study, therefore, was designed to determine the effect of TGF-β1 on fibroblast apoptosis in contracting three-dimensional collagen gels. TGF-β1 was found to stimulate both contraction of collagen gels and the apoptosis of fibroblasts in contracting gels. This contrasted with a slight inhibition of apoptosis in fibroblasts in three-dimensional gels that were constrained from contracting. It also contrasted with the effect of serum and PDGF, which stimulated contraction without stimulating apoptosis. These results, therefore, suggest that TGF-β1 may stimulate contraction of fibroblasts which, in turn, may lead to fibroblast apoptosis. Such a coordinated action may be a key feature of normal tissue repair by preventing the persistent accumulation of fibroblasts within tissues. These findings suggest that growth factors other than TGF-β may contribute to the contraction with persistence of fibroblasts that is noted in fibrotic tissues.

## Methods

### Materials and cell culture

Type I Collagen (rat tail tendon collagen [RTTC]) was extracted from rat-tail tendons by a previously published method [16]. Protein concentration was determined by weighing a lyophilized aliquot from each batch of collagen. The RTTC was stored at 4°C until use. Dulbecco's modified Eagle's medium (DMEM), fetal calf serum (FCS), trypsin/EDTA, penicillin G sodium, and streptomycin were purchased from Invitrogen (Life Technologies, Grand Island, NY). Amphotericin B was purchased from Pharma-Tek (Elmira, NY). The terminal transferase dUTP nick end labeling (TUNEL) assay kit was purchased from Roche Diagnostic Corporation (Indianapolis, IN). Goat anti-caspase 3 antibody (CRP32), which reacts with both precursor and active forms of human caspase 3, and goat anti-PARP, which reacts with both intact and cleaved forms of human PARP, rabbit anti-cIAP-1 antibody, mouse anti-XIAP antibody, recombinant human TGF-β1, PDGF-BB and anti-TGF-β1 antibody were purchased from R&D Systems (Minneapolis, MN). Mouse anti-Bcl-2 antibody and mouse anti-Bax antibody were purchased from Santa Cruz Biotechnology, Inc. (Santa Cruz, CA). Rabbit anti-goat and mouse IgG horseradish peroxidase were purchased from Rockland Immunochemicals (Gilbertsville, PA). Propidium iodide, staurosporine and anti-β-actin antibody were purchased from Sigma (St. Louis, MO).

Human fetal lung fibroblasts (HFL-1) were obtained from the American Type Culture Collection (Rockville, MD). Smad2 knockout and corresponding wildtype, and Smad3 knockout and corresponding wildtype were kind gifts from Dr. A. Roberts (NIH). The Smad2 knockout (S2KO) mouse fibroblasts were established from mouse embryo-derived fibroblasts harboring the null allele Smad2^Δex2 ^in the homozygous state, as described [17,18]. Smad3 knockout (S3KO) mice were generated by targeted deletion of exon 8 in the Smad3 gene by homologous recombination, as described [18,19]. The cells were cultured in 100-mm tissue culture dishes (Falcon; Becton-Dickinson Labware, Lincoln Park, NJ) in Dulbecco's Modified Eagle's Medium (DMEM), supplemented with 10% fetal calf serum (FCS), 50 U/ml penicillin G sodium, 50 μg/ml streptomycin sulfate, and 1 μg/ml amphotericin B. The fibroblasts were refed three times weekly, and cells between passages 15 to 18 for human and 34 to 45 for murine were used.

Small interfering RNA (siRNA) for Smad3 was designed to target the coding sequence of human Smad3 and effectively inhibits Smad protein expression as described previously [20]. siRNA for Smad2 and non-specific siRNA for control were purchased from Dharmacon (SMARTpool). Transfection of siRNA was also performed as described previously [20]. After 24 hours transfection, HFL-1 cells were harvested and used for gel contraction assay.

### Three-dimensional collagen gel culture

Prior to preparing collagen gels as described below, fibroblasts were detached by 0.05% trypsin in 0.53 mM EDTA and suspended in 10 ml serum-free DMEM containing soybean trypsin inhibitor. The cell number was then counted with Coulter Counter. Collagen gels were prepared, as previously described [16], by mixing RTTC, distilled water, 4 × DMEM and cells. The final concentration was 1 × DMEM, 0.75 mg/ml of collagen, and fibroblasts were present at 3 × 10^5 ^cells/ml for human and 4.5 × 10^5 ^cells/ml for murine. Following this, 500 μl of the mixture was cast into each well of a 24-well culture plate (Falcon). The solution was then allowed to polymerize at room temperature, generally completed in 20 min. After polymerization, the gels were either allowed to remain attached to the plates in which they were case or, for the gel contraction assay, the gels were gently released from the plates in which they were cast and transferred into 60-mm tissue culture dishes (three gels in each dish), which contained 5 ml of SF-DMEM with or without FCS, TGF-β1 and PDGF-BB, respectively. The concentrations of TGF-β1 used were based on previous studies [21,22]. The area of each gel was measured daily with an image analyzer (Optomax, Burlington, MA). Data are expressed as the percentage of area compared with the initial gel area. For attached gels, gels were left attached in the plates and 1 ml of SF-DMEM with or without FCS or TGF-β1 was added. The gels were then incubated at 37°C in a 5% CO_2 _atmosphere.

### DNA quantification

To estimate cell number in three-dimensional collagen gels, DNA was assayed fluorometrically with Hoechst dye no. 33258 (Sigma) by a modification of a previously published method [23]. Collagen gels were solubilized by heating to 60°C for 10 min and cell suspensions were collected by centrifugation at 2,000 × *g *for 5 min and resuspended in 1 ml of distilled water. After sonication, the suspensions were mixed with 2 ml of TNE buffer (3 M NaCl, 10 mM Tris, and 1.5 mM EDTA, pH7.4) containing 2 μg/ml of Hoechst dye no. 33258. Fluorescence intensity was measured with a fluorescence spectrometer (LS-5; Perkin-Elmer, Boston, MA) with excitation at 356 nm and emission at 458 nm.

### Determination of apoptosis (TUNEL assay)

For determination of apoptosis, TUNEL assay was performed following manufacturer's instructions. Briefly, collagen gels were transferred from medium or plates attached to Eppendorf tubes (Fisher, Pittsburgh, PA) and then solubilized with heating at 60°C for 10 min. This method effectively solubilized the collagen gels without resulting in further DNA damage, as assessed by TUNEL assay (data not shown). Cell suspensions were collected by centrifugation at 2,000 × *g *for 5 min and resuspended in 150 μl of 10% FCS-DMEM. The resuspended cells were then used to prepare cytospins, 0.5 × 10^5 ^cells/spot, 1,000 × *g *for 5 min. Cytospin preparations were fixed with freshly prepared paraformaldehyde (4% in phosphate-buffered saline [PBS]; pH 7.4) for 1 h at room temperature. The cells were permeabilized with 0.1% Triton X-100 (in 0.1% sodium citrate) for 2 min at 4°C and rinsed with PBS. The TUNEL reaction was then performed using the manufacturer's instructions (Roche). The number of cells stained by the TUNEL method was expressed as a percentage of the total number of cells stained with the counterstain propidium iodide. At least 500 nuclei were counted on each cytospin sample in 5–10 randomly selected viewing fields.

### Profile of DNA content by flow cytometry

For three-dimensional collagen gel culture, DNA content was analysed as described [24]. Briefly, fibroblast-populated (2 ml of 3 × 10^5^cells/ml) collagen gels were cast into 6-well tissue culture plates (Falcon). After polymerization, gels were gently released and incubated with 1 % FCS-DMEM for 24 h, 100 pM TGF-β1 or with 1 μM staurosporine for 6 h (positive control). Gels were then transferred into 15-ml conical tubes and incubated with 0.05% Trypsin/0.53 mM EDTA-4Na (Invitrogen) for 10 min (500 μl/gel) at 37°C in a 5% CO_2 _atmosphere. Collagenase (1 mg/ml in DMEM) was then added (1 ml/gel) and incubated while shaking at 37°C in a 5% CO_2 _atmosphere for 30 min or until the gels were completely dissolved. DMEM containing 10% FCS was then added to stop the enzymatic reaction, and cells were pelleted by centrifugation. Cells were then fixed with Telford method and flow cytometry was performed as described below.

Flow cytometric analysis of DNA content was performed as previously described [25]. Briefly, cells were fixed with cold 70% ethanol in PBS for 30 min at 4°C. Cells were then pelleted by centrifugation and resuspended in the staining solution (50 μg propidium iodide, 100 μg RNAse A in 1 ml PBS for 10^6 ^cells) at 4°C for 1 h followed by flow cytometric analysis without washing. Since harvesting cells from the gels at day 5 results in formation of considerable debris which made the DNA profiling assay problematic, we chose day 1 for DNA profiling.

### Western blot analysis

Three-dimensional collagen gel culture was performed as described above. After collecting cells by centrifugation, cells were washed with sterile PBS twice, and then put 100 μl cell lysis buffer (35 mM Tris-HCl, pH 7.4, 0.4 mMEGTA, 10 mM MgCl_2_, 100 μg/ml aprotinin, 1 μM phenylmethylsulfonyl fluoride, 1 μg/ml leupeptin, and 0.1% Triton X-100). Lysates were briefly sonicated on ice and centrifuged at 10,000 g for 3 minutes. The protein concentration in the cell lysates was measured using the BIO-RAD Protein Assay Kit. 10% SDS-polyacrylamide gel electrophoresis was performed under reducing conditions. To accomplish this, cell lysate proteins were diluted with 2× concentrated sample buffer (250 mM Tris-HCl, pH 6.9, 4% SDS, 10% glycerol, 0.006% bromphenol blue, 2% β-mercaptoethanol) and heated at 95°C for 5 minutes before loading (10 μg/lane). After SDS-PAGE, proteins were transferred onto PVDF membrane (BIO-RAD). The membrane was blocked for 1 h at room temperature with 5% skim milk in PBS-Tween and incubated overnight at 4°C with proper each antibody concentrations, respectively. After incubation with HRP-conjugated anti-Rabbit or mouse-IgG, an ECL Western blot detection system was used according to the manufacture's instruction (Amersham Biosciences, Piscataway, NJ).

### Statistical analysis

Results are presented as mean ± SEM. Statistical comparison of paired data was performed using Student's *t *test, whereas multigroup data were analyzed by ANOVA followed by the Tukey's or Bonferroni's post-test using Statview software (Abacus Concepts Inc., Cary, NC). *P *< 0.05 was considered significant.

## Results

### Effect of FCS and TGF-β1 on fibroblast-mediated collagen gel contraction

**Figure 1:**
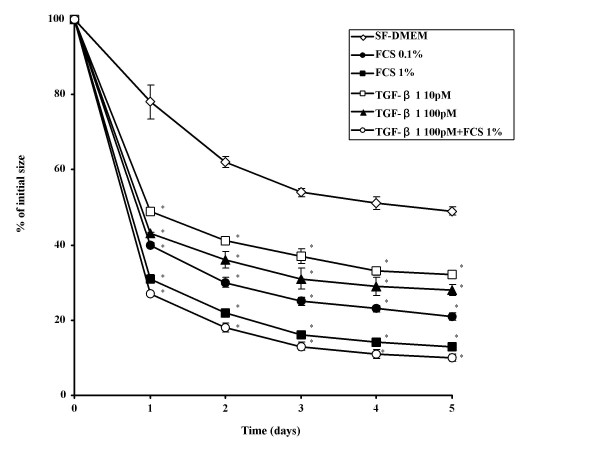
**Effect of TGF-β1 and FCS on collagen gel contraction mediated by HFL-1 cells. **Fibroblast-populated collagen gels were released into 60 mm tissue culture dishes with or without FCS or TGF-β1. Gel size was measured daily with an image analyzer. Vertical axis: gel size expressed as % of initial size. Horizontal axis: Time (days of culture). Both serum and TGF-β1 significantly augmented collagen gel contraction in a concentration-dependent manner. *p < 0.05 as compared with control. Data are shown as means ± SEM. Data presented are from one representative experiment of three experiments performed on separate occasions.

Both FCS and TGF-β1 increased the contraction of collagen gels in a concentration-dependent manner over the period of observation. After 5 days, control gels (SF-DMEM) were 50.0 ± 1.1% of their initial area (Figure 1). In contrast, gels exposed to FCS (0.1% or 1%) were 21.6 ± 1.0% and 13.1 ± 0.1% of their original size after 5 d, respectively (Figure 1). Gels exposed to TGF-β1 (10 pM or 100 pM) were 32.8 ± 0.5% and 28.8 ± 1.5% of their original size after 5 d, respectively (Figure 1). The effect of FCS and TGF-β1 were both concentration- and time-dependent. Addition of anti-TGF-β antibodies did not alter the effect of serum but did completely block the effect of TGF-β (data not shown).

### Effect of FCS and TGF-β1 on apoptosis

**Figure 2:**
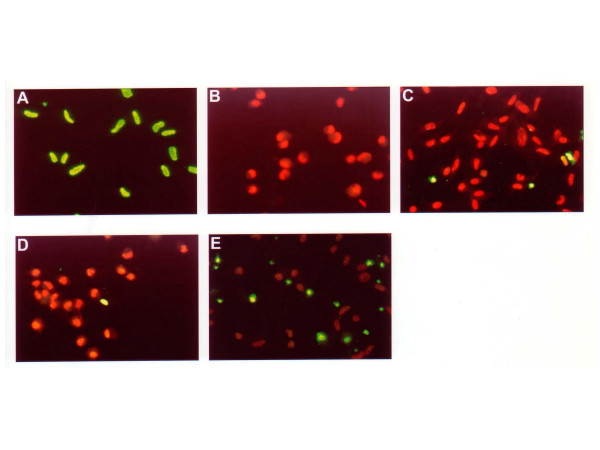
**TUNEL staining in HFL-1 cells. **Fibroblast-populated collagen gels were released into 60 mm tissue culture dishes with or without FCS or TGF-β1. On day 5, collagen gels were digested, cells isolated, cytocentrifuge preparation made, and stained by TUNEL. A: positive control (DNAse-1 treated), B: negative control (without terminal transferase), C: FCS free, D: FCS 1%, E: TGF-β1(100 pM). Red: PI stained normal cells. Green: TUNEL positive cells. Data presented are from one representative experiment. Similar results were obtained in three experiments performed on separate occasions.

**Figure 3:**
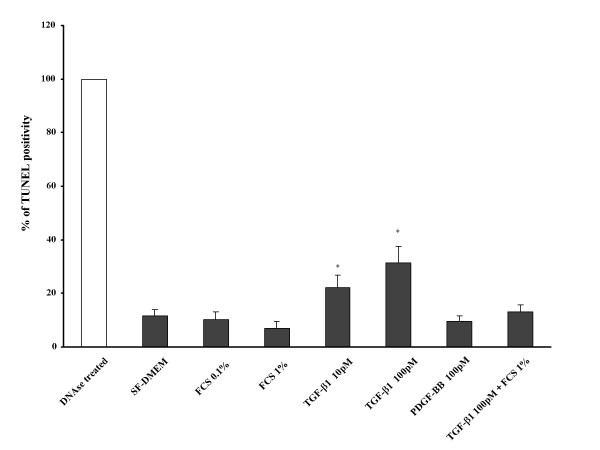
**TUNEL positivity in HFL-1 with FCS, TGF-β1 and PDGF-BB. **After staining, TUNEL positive cells as a % of total cells were counted under the microscope in 5 high-power fields. Vertical axis: TUNEL positivity expressed as % of positive control (DNAse treated). Horizontal axis: condition. TGF-β1 increased TUNEL positivity. In contrast, FCS or PDGF-BB did not affect TUNEL positivity. *p < 0.05, as compared with control. Data are shown as means ± SEM. Data presented are from one representative experiment of three experiments performed on separate occasions.

To determine the effect of FCS and TGF-β1 on fibroblast apoptosis, two methods were used. First, cells in three-dimensional collagen gels were cultured in SF-DMEM, 0.1% or 1%FCS-DMEM, 10 pM or 100 pM TGF-β1, and as an additional comparator 100 pM PDGF-BB for 5 days, and then TUNEL staining which measures DNA strand breaks, a feature of apoptosis cells, was performed (Figure 2). After 5 days, 11.6 ± 0.3% of control cells were TUNEL positive (Figure 3). TGF-β treated cells had increased TUNEL positivity while FCS treated cells had decreased TUNEL positivity. To quantify this, 500 cells from each condition were counted. In the presence of 0.1% FCS or 1% FCS, 10.3 ±0.5% and 7.1 ± 0.9% of the cells were TUNEL positive, respectively (Figure 3). PDGF-BB (100 pM) stimulated gel contraction similarly to TGF-β1 (data not shown) but did not result in increased apoptosis above control, 10.8 ± 0.4% of the PDGF-BB treated cells were TUNEL positive. In contrast, in the presence of 10 pM or 100 pM TGF-β1, TUNEL positive cell numbers were significantly increased to 22.3 ± 0.4% and 31.4 ± 1.4%, respectively (Figure 3) (p < 0.05, compared with control).

**Figure 4:**
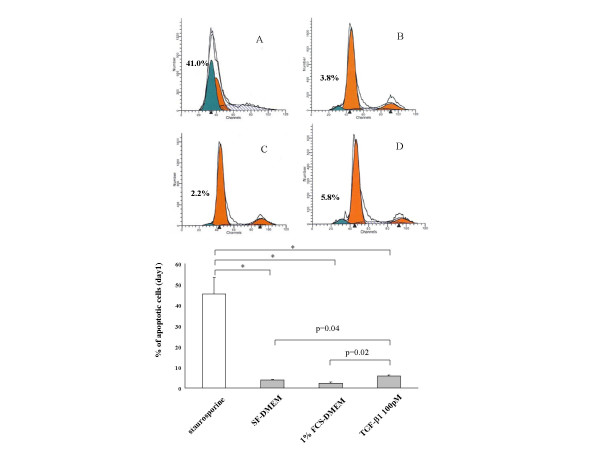
**Representative profile of DNA content in fibroblasts. **Collagen gels with fibroblasts were floated in (A) Staurosporine 1 μM for 6 hours, (B) SF-DMEM, (C) 1% FCS-DMEM and (D) TGF-β1 100 pM for 24 hours. Cells were then isolated and analyzed by flow cytometry. *Vertical axis*: cell number; *horizontal axis*: DNA content. The percentage of cells with hypodiploid DNA taken as an index of apoptosis is shown in each panel. Figure presented is from one representative experiment of three experiments performed on separate occasions. *p < 0.01. Data are shown as means ± SEM. Comparison of the means were done by one-way ANOVA.

To confirm the presence of apoptosis, profiling of DNA content was performed by flow cytometry. As a positive control, a group of gels were treated with staurosporin. After 24-hours, 1% FCS had tendency to decrease the amount of hypodiploid DNA compared to control cultures. In contrast, the TGF-β1 group increased the amount of hypodiploid DNA compared to control, indicating TGF-β1 increased apoptosis while FCS did not (Figure 4).

**Figure 5:**
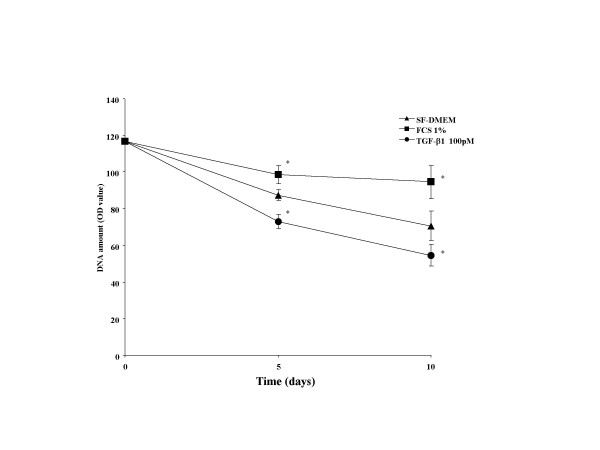
**DNA amount in contracting collagen gels. **Fibroblasts were embedded in collagen gels and cultured in floating media containing 1% FCS or 100 pM TGF-β1 or control. DNA content, as a surrogate for cell number, was determined at the time of plating and after 5 and 10 days. *P < 0.05, as compared with SF-DMEM. Data are shown as means ± SEM.

### Time course of cell numbers in three-dimensional collagen gel

To further confirm that apoptosis was occurring, the DNA amount, which can be used as a surrogate for cell number, was assessed in floating collagen gels. After casting gels in the presence of either serum-free DMEM, 1% fetal calf serum or 100 pM TGF-β1, DNA amount was assessed after 5 and after 10 days without further refeeding. As expected, DNA content decreased over time in control cultures incubated in DMEM alone. In the presence of 1% FCS, DNA amount decreased, but the decrease was statistically significantly less than that which occurred under control conditions (p < 0.05). In contrast, in the presence of TGF-β1, the decrease in DNA amount was larger than that which occurred in control (p < 0.05).

### Effect of FCS and TGF-β1 on apoptosis related protein expression

**Figure 6:**
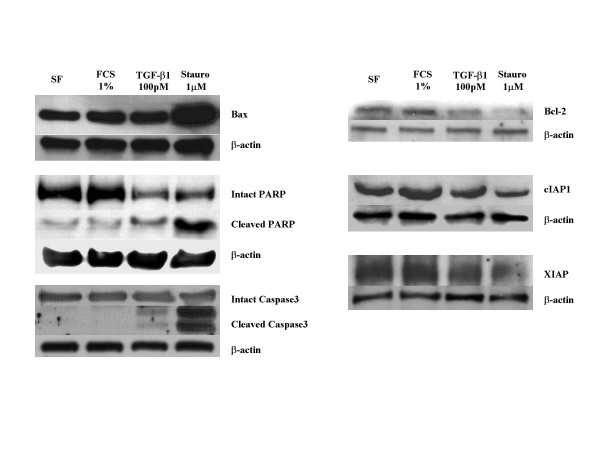
**Western blots of selected pro-apoptotic and anti-apoptotic factors. **Fibroblasts were embedded in collagen gel and cultured in floating media with 1% FCS, 100 pM TGF-β1, staurosporine or control. After a day, collagen gels were digested, cells were collected, lysed and the cell lysate evaluated by Western blot. Data presented are from one representative experiment. Similar results were obtained in three experiments performed on separate occasions.

A large number of proteins can serve as positive or negative regulators of the apoptosis process. To further confirm the differential effect of fetal calf serum and TGF-β1 on apoptosis, several apoptosis-related proteins were evaluated by Western blot (Figure 6). Staurosporin, which is an active control and induced apoptosis, increased the expression of Bax and induced the cleavage of both PARP and caspase 3, three markers of active apoptosis while it simultaneously inhibited the expression of Bcl-2, cIAP-1 and XIAP, three inhibitors of apoptosis. In contrast to the effects of staurosporin, 1% FCS stimulated the expression of Bcl-2, cIAP-1 and XIAP, the inhibitors of apoptosis, while it resulted in no cleavage of PARP or caspase 3. TGF-β1, in contrast, resembled staurosporin by increasing the expression of Bax and initiating the cleavage of PARP and caspase 3, all markers of active apoptosis, while it simultaneously inhibited the expression of Bcl-2 and XIAP.

### Effect of FCS and TGF-β1 on apoptosis in the attached gels

**Figure 7:**
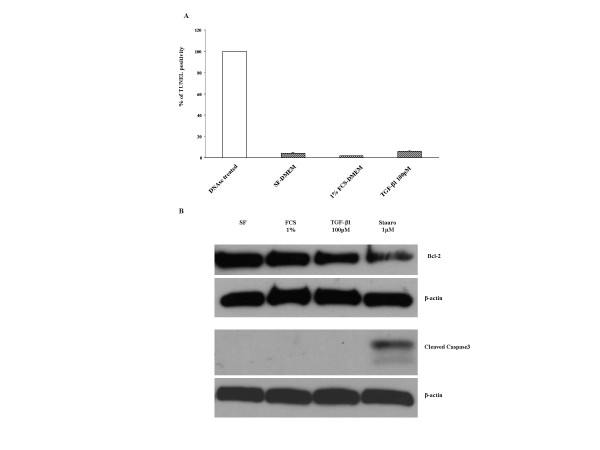
**TUNEL positivity in HFL-1 cells cultured in attached gels and Western blots for selected pro-apoptotic and anti-apoptotic factors. **TUNEL Positivity. (A) Fibroblasts embedded in collagen gels which were left attached to the plates preventing contraction. After 5 days, gels were digested and stained for TUNEL. TUNEL positive cells were counted in 5 high-power fields and expressed as % of total cells. Data are presented as % of positive control (DNAse treated). Data are shown as means ± SEM. Western blot for selected pro-apoptotic and anti-apoptotic factors. (B) Collagen gels were digested, cells were collected, lysed and the cell lysate were evaluated by Western blot. Data presented are from one representative experiment.

To determine if the effect of FCS and TGF-β1 on fibroblast apoptosis in collagen gels was related to contraction, cells in three-dimensional collagen gels were cultured in SF-DMEM, 1% FCS-DMEM or 100 pM TGF-β1 for 5 days and the gels were left attached to the plates, which prevents contraction. After this, the cultures were harvested and TUNEL staining was performed (Figure 7A). In contrast to contracting gels, 100 pM TGF-β1 did not significantly increase the percentage of TUNEL positive cells in attached gels. Similarly, in contrast to the effect on floating gels, TGF-β exposure had no effect in activating caspase 3 in gels that were constrained from contracting (Figure 7B).

### Role of Smad2 and Smad3 in TGF-β induced apoptosis of fibroblasts in floating collagen gels

**Figure 8:**
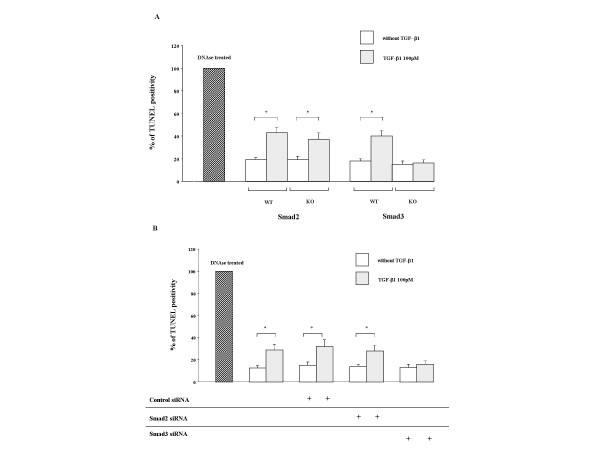
**TUNEL positivity and Western blot of selected pro-apoptotic and anti-apoptotic factors in murine fibroblasts and HFL-1 cells with or without TGF-β1. **After staining, TUNEL positive cells as a % of total cells were counted under the microscope in 5 high-power fields. Panel A: Murine Smad3 KO and control cells; Panel B: HFL-1 cells ± siRNAs. Vertical axis: TUNEL positivity expressed as % of positive control (DNAse treated). Horizontal axis: condition. TGF-β1 increased TUNEL positivity in all cell types except in S3KO cells (Panel A) and Smad3 siRNA cells (Panel B). *p < 0.05, as compared with control. Data are shown as means ± SEM. Data presented are from one representative experiment of three experiments performed on separate occasions.

**Figure 9:**
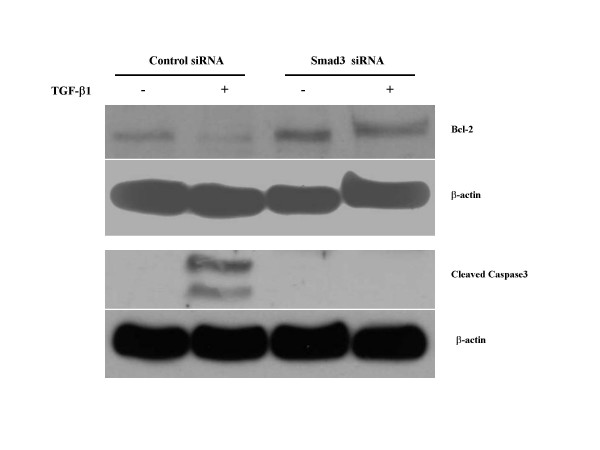
**Western blot for selected pro- and anti-apoptotic proteins in HFL-1 cells treated with Smad3. **Collagen gels made from Smad siRNA cells and control siRNA cells were digested, cells were collected, lysed and the cell lysate were evaluated by Western blot. Data presented are from one representative experiment repeated twice.

To determine the role of Smad2 and Smad3 on fibroblasts apoptosis, two methods were used. Murine lung fibroblasts from S2KO and S3KO and the corresponding wildtype (S2WT and S3WT) and HFL-1 cells incubated with siRNA targeting Smad2 and Smad3 were cultured in 3-D collagen gels with or without TGF-β1. As expected, TGF-β1 did not induced augmented contraction in Smad3 KO cells as previously described [11] or in Smad3 siRNA treated HFL-1 cells (data not shown). In contrast, TGF-β1 significantly augmented contraction in Smad2 KO cells in both wildtype controls [11] and in Smad2 siRNA treated and control HFL-1 cells (data not shown). After 5 days, TUNEL staining was performed. S2KO cells and both types of wildtype control cells as well as Smad2 siRNA treated and control HFL-1 cells had increased TUNEL positivity after TGF-β1 treatment (Figure 8). In contrast, TGF-β1 had no effect on TUNEL positivity in either Smad3 knockout mouse or Smad3 siRNA treated HFL-1 cells. Similarly, TGF-β did not result in the activation of caspase 3 in Smad3 siRNA treated HFL-1 cells (Figure 9).

## Discussion

The current study evaluated the survival of fibroblasts in contracting three-dimensional collagen gels. As expected, TGF-β1, PDGF-BB and serum all stimulated fibroblast-mediated contraction of three-dimensional collagen gels. TGF-β1 also stimulated apoptosis in the fibroblasts as assessed by both TUNEL assay and confirmed by DNA profiling to quantify cells with hypodiploid DNA content. In contrast, neither fetal calf serum nor PDGF-BB altered fibroblast apoptosis in contracting collagen gels. The stimulatory effect of TGF-β1 on apoptosis was associated with an increase in pro-apoptotic markers, including cleaved caspase 3, Bax and cleaved PARP, as well as inhibition of anti-apoptotic factors, including Bcl-2, cIAP-1 and XIAP. The ability of TGF-β1 to stimulate apoptosis required contraction of the three-dimensional collagen gels as no induction of apoptosis was noted in gels that were constrained from contraction.

TGF-β1 is one of three TGF-β isoforms that are members of a family of signaling molecules [5] TGF-β1 is believed to be a key factor in a variety of physiological and disease processes mediating a diverse range of cellular responses, including down regulation of inflammation, stimulation or inhibition of various cells types and regulation of differentiation of many target cells. TGF-β1 is believed to play a particularly important role as a mediator of wound healing [6]. TGF-β1 is a potent activator of fibroblasts stimulating fibroblast proliferation, production of extracellular matrix and differentiation into myofibroblasts. Because of these actions, TGF-β1 driven fibroblast activation is believed to play a major role in wound repair, scar formation and tissue fibrosis [26,27].

Tissue fibrosis differs from normal wound repair in several important features. While both are characterized by proliferation and accumulation of fibroblasts together with the extracellular matrix produced by these cells, normal granulation tissue is characterized by a resolution phase [28]. Specifically, as granulation tissue contracts, fibroblast apoptosis together with resorption of some of the collagenous extracellular matrix characteristically takes place. In fibrotic tissues, the severity of scarring and fibrosis, therefore, is dependent not only on the degree of fibroblast activation, but also on the relative lack of resolution. While the mechanisms that regulate resolution are incompletely understood, the current study supports the concept that TGF-β1 can drive fibroblast apoptosis concurrent with tissue contraction and that TGF-β1 differs from other growth factors in this regard. These results, which were obtained with fibroblasts cultured in three-dimensional collagen gels, contrast markedly with previous studies that evaluated fibroblasts cultured in monolayer culture where TGF-β inhibits apoptosis.

The members of the TGF-β family signal through a family of receptors, the activin receptors, which in turn signal through a family of signal transduction molecules, the Smads [29]. TGF-β signals primarily through the TGF-β RII (activin IIB) which phosphorylates the TGF-β RI (activin I). The activin I receptor, in turn, phosphorylates two Smad proteins, Smad 2 and Smad 3, which subsequently bind Smad 4 and mediate TGF-β signaling. While these represent the best characterized mechanisms for TGF-β signaling, other signaling pathways independent of Smad 2 and 3 have been reported [30]. The concentrations of TGF-β used in the current study were based on previous in vitro studies and are in the range expected for TGF-β to be active on its receptor. In vivo concentrations of TGF-β have been measured and are generally many-fold greater than those used. In vitro measurements, however, have generally assessed total TGF-β rather than the active form. Thus, while measures of in vivo active TGF-β concentrations are unavailable, the concentrations used in the current study are likely to be biologically relevant.

The culture of fibroblasts in three-dimensional collagen gels has been used for several decades as a model of tissue contraction that characterizes wound healing [1]. When cultured in floating collagen gels, fibroblasts attach to the collagenous matrix through integrin-dependent mechanisms and exert mechanical tension, which can cause floating gels to contract. In addition, concurrent with contraction, fibroblasts undergo apoptosis [13-15]. Interestingly, the amount of apoptosis is related to the amount of contraction [13,14]. Gels prepared with smaller concentrations of collagen, for example, undergo greater degrees of contraction, and a higher percentage of fibroblasts undergo apoptosis [14]. While the mechanisms that regulate apoptosis under these conditions are not fully established, cell spreading may play a role [31]. Specifically, cells that are not effectively spread are susceptible to apoptosis. Contraction, therefore, may be related to apoptosis induction. An effect of mechanical tension may also play a role. Finally, although our results suggest that contraction, per se, is related to induction of apoptosis, it is possible that other effects of TGF-β that also depend on Smad3 signaling mediate this effect.

Fibroblasts cultured in collagen gels can also proliferate. However, their response to growth factors in gel culture can be attenuated. Under the conditions used in the current assay, we have previously shown that there is minimal stimulation of proliferation with serum concentration 1% or less [16]. Serum contains many factors that can inhibit apoptosis [32], although the factors involved remained to be defined. Whether serum stimulation of contraction results from the same factor(s) that block apoptosis remain to be determined, although PDGF can do both. The overall effect of serum, however, contrasts with that of TGF-β. The link between TGF-β induced contraction and apoptosis may be a mechanism to prevent the accumulation of fibroblasts in resolving wounds. In contrast, the persistence of fibroblasts induced by other factor(s) present in serum may be a mechanism that contributes to scar formation or fibrosis.

The key finding of the current study is that augmented contraction induced by TGF-β is associated with apoptosis. This contrasts with augmented contraction induced by either PDGF or serum that is not associated with augmented apoptosis. These results suggest that contraction that takes place in the presence of TGF-β can be associated with apoptosis of fibroblasts. While TGF-β has been suggested to be a ''pro-fibrotic'' mediator because of its frequent association with both tissue injury and repair and with fibrotic processes and with its ability to activate fibroblasts, the present study suggests that TGF-β may stimulate fibroblasts in such a way that ''resolution'' is possible. The failure of apoptosis to occur in the presence of augmented contraction induced by PDGF and serum, however, suggests that other growth factors, that could function in collaboration with TGF-β, may be responsible for the persistence of fibroblasts and, hence, the development of fibrosis.

In order to determine the mechanisms by which TGF-β signaling leads to apoptosis, two approaches were used. TGF-β signaling was suppressed using siRNAs for either Smad 2 or Smad 3 and fibroblasts cultured from Smad 2 or Smad 3 deficient mice were compared with appropriate controls. As previously described [11], the absence of Smad 2 signaling had no effect on TGF-β1 or PDGF-BB stimulation of collagen gel contraction, while the absence of Smad 3 signaling blocked the ability of TGF-β1 to augment contraction, but not the ability of PDGF-BB to augment contraction. Using both siRNA and genetically deficient mice, loss of Smad 2 signaling had no effect on TGF-β1 augmentation of apoptosis, while loss of Smad 3 signaling blocked the ability of TGF-β1 to augment apoptosis. Thus, inhibition of apoptosis was always associated with inhibition of contraction.

The effect of TGF-β contrasted with the effect of serum which augmented contraction but did not stimulate apoptosis. These differing effects on apoptosis were paralleled by effects on apoptosis-related proteins. The mechanisms that prevent apoptosis in the presence of serum (or PDGF-BB) are unclear. In the present study, neither PDGF-BB nor serum affected apoptosis in a statistically significant manner. However, a small inhibition of apoptosis that did not achieve statistical significance was observed. Thus, it is possible that PDGF-BB or other growth factors could actively suppress apoptosis. In this context, the presence of serum was associated with an increase in cIAP-1 and Bcl-2, anti-apoptotic proteins. Interestingly, serum was also associated with an apparent increase in Bax, a pro-apoptotic protein. It seems likely, therefore, that factors present in serum may be able to affect the balance between pro- and anti-apoptotic factors and through such mechanisms could stimulate contraction while inhibiting apoptosis.

Apoptosis, or programmed cell death, is a highly regulated intracellular process. It can be initiated through several signaling mechanisms, including both activation of specific receptors as well as through non-specific effects such as DNA damage [33-35]. Apoptosis is regulated at several levels. Important among these is the proteolytic caspase cascade [36]. The caspases form a series of enzymatic reactions that, through successive cleavage events, can lead to the activation of caspase 3 which functions as a "cellular executioner." Concurrently, proteolytic cleavage can degrade the enzyme PARP which serves to maintain DNA integrity. The cleavage of PARP, an enzyme that mediates DNA repair, is believed to be an early step that commits a cell to death rather than DNA repair [37,38]. Similarly, cleavage of caspase 3 to its active form is believed to be a step that commits a cell to apoptosis as caspase 3 subsequently degrades many key cellular proteins. The commitment of a cell to apoptosis, therefore, can be regulated by controlling the activity of caspases. Several mechanisms exist by which this can be accomplished, including the release of the co-factor cytochrome C from mitochondria [39], which is both positively and negatively regulated by members of the Bax/Bcl family and by regulation through a family of inhibitors of caspases [40]. In this context, TGF-β1 induction of apoptosis in contracting three-dimensional collagen gels was associated with augmented expression of Bax, a pro-apoptotic member of the Bax/Bcl-2 family together with inhibition of Bcl-2, an anti-apoptotic member of the same family. Similarly, TGF-β1 was associated with inhibition of both cIAP-1 and XIAP, two inhibitors of the caspase cascade. The mechanisms by which TGF-β induces these effects are beyond the scope of the current proposal, but appears to require contraction of the three-dimensional collagen gels. This raises the possibility that the effect of TGF-β is indirect and may be related to cell spreading, for example.

In conclusion, the current study demonstrates that TGF-β1 induction of three-dimensional collagen gel contraction is associated with apoptosis. This induction of apoptosis requires contraction of the three-dimensional collagen gels and differs from other factors, including serum and PDGF-BB that induce contraction but not apoptosis. The ability of TGF-β to induce apoptosis may play a key role during wound repair. Abnormal regulation of apoptosis during the resolution phase following tissue repair could contribute importantly to both hypertrophic scar formation as well as to tissue fibrosis. The ability of tissues to contract normally may be important in this regard, and processes that increase mechanical tension in tissues or constrain contraction by other mechanisms may contribute to fibrosis and tissue remodeling. This study, therefore, supports the concept that TGF-β induction of fibroblast apoptosis is one of its many functions related to tissue repair and remodeling. Alterations in this function could contribute to the formation of hypertrophic scar or tissue fibrosis.
